# Lineage-Specific Gene Family Innovations Underpin the Pathogenicity of Woody-Plant Pathogens in Botryosphaeriaceae

**DOI:** 10.1093/gbe/evag178

**Published:** 2026-07-17

**Authors:** Xuncheng Wang, Junbo Peng, Linna Wu, Xinghong Li, Wei Zhang, Jiye Yan

**Affiliations:** Beijing Key Laboratory of Environment Friendly Management on Fruit Diseases and Pests in North China, Institute of Plant Protection, Beijing Academy of Agriculture and Forestry Sciences, Beijing 100097, China; Beijing Key Laboratory of Environment Friendly Management on Fruit Diseases and Pests in North China, Institute of Plant Protection, Beijing Academy of Agriculture and Forestry Sciences, Beijing 100097, China; Beijing Key Laboratory of Environment Friendly Management on Fruit Diseases and Pests in North China, Institute of Plant Protection, Beijing Academy of Agriculture and Forestry Sciences, Beijing 100097, China; Beijing Key Laboratory of Environment Friendly Management on Fruit Diseases and Pests in North China, Institute of Plant Protection, Beijing Academy of Agriculture and Forestry Sciences, Beijing 100097, China; Beijing Key Laboratory of Environment Friendly Management on Fruit Diseases and Pests in North China, Institute of Plant Protection, Beijing Academy of Agriculture and Forestry Sciences, Beijing 100097, China; Beijing Key Laboratory of Environment Friendly Management on Fruit Diseases and Pests in North China, Institute of Plant Protection, Beijing Academy of Agriculture and Forestry Sciences, Beijing 100097, China

**Keywords:** Botryosphaeriaceae, gene family expansion, gene family contraction, pathogenicity

## Abstract

Botryosphaeriaceae is an important fungal family that colonizes a wide range of woody hosts, including economically important crops, and causes serious diseases worldwide. This family has undergone substantial genomic innovations during its evolutionary history. However, the role of gene family innovations, particularly gene contractions, in shaping pathogenicity within Botryosphaeriaceae remains unclear. To address this, we generated five high-quality genomes from three species representing three genera of Botryosphaeriaceae and combined them with four previously published genomes. These nine genomes were analyzed in a comparative genomic framework together with 47 publicly available fungal genomes. Multiple rounds of gene family expansions were identified in Botryosphaeriaceae, including ancient expansions that occurred prior to the divergence of genera and genus-specific expansions that arose afterward. These expansions were associated with genes involved in host infection and virulence. In contrast, gene family contractions were less frequent but were also associated with functions potentially related to pathogenicity when they occurred after the divergence of Botryosphaeriaceae into distinct genera. Transcriptomic analysis of *Lasiodiplodia theobromae* further supported the functional significance of lineage-specific gene family expansions and contractions in virulence evolution. Silencing a lineage-specific expanded gene encoding a plant cell wall-degrading enzyme from the GH11 family significantly reduced the virulence of *L. theobromae*, confirming its role in pathogenicity. Together, these findings reveal that lineage-specific gene family innovations, including both expansions and contractions, have driven virulence diversification and adaptation among Botryosphaeriaceae species during host–pathogen interactions.

Significance Gene family expansion and contraction are common features of phytopathogenic fungi. However, how these gene family innovations, particularly contractions, shape the evolution of pathogenicity remains unclear. Focusing on the Botryosphaeriaceae, a globally distributed family of woody plant pathogens that has undergone extensive gene family turnover, we compared the genomes of nine strains representing three genera with distinct virulence levels. Our analyses reveal that gene family expansions are prevalent in Botryosphaeriaceae and are broadly associated with the evolution of pathogenicity, whereas gene family contractions, although less frequent, may also contribute to virulence evolution in a lineage-specific manner. Together, our findings demonstrate that both gene family expansions and lineage-specific contractions represent recurrent evolutionary mechanisms driving pathogenic diversification within Botryosphaeriaceae.

## Introduction

The evolution of fungal pathogenicity is driven by continuous genetic innovation that enables pathogens to colonize, exploit, and adapt to diverse hosts. Among these evolutionary processes, lineage-specific expansion and contraction of gene families, particularly those involved in plant cell wall degradation, secondary metabolism, and host interaction, have been recognized as key mechanisms shaping fungal virulence and host range (Dou and Zhou 2012; O'Connell et al. 2012; Hartmann and Croll 2017; Rogers et al. 2022). Despite increasing evidence that such gene family changes contribute to host specialization and pathogenic diversification, their precise evolutionary roles remain incompletely understood.

Botryosphaeriaceae (Dothidiomycetes, Ascomycota) represents an ideal system to investigate how gene family innovations underpin pathogenicity and host adaptation. This fungal family colonizes a wide range of woody plants worldwide and comprises 24 well-defined genera and more than 200 species (Burgess et al. 2019). Most of these species are classified as pathogens that can cause disease on a wide range of economically important plants (Yan et al. 2013; Delgado-Cerrone et al. 2016; Mancero-Castillo et al. 2018; Feijo et al. 2019). Members of Botryosphaeriaceae typically infect host plants via wounds or natural openings and colonize the bark surface and internal woody tissues without causing any obvious symptoms for a long period, which may be considered as an “endophytic phase” before the onset of severe disease symptoms (Yan et al. 2013; Morales-Cruz et al. 2015). This quiescent phase may be disrupted by abiotic stresses such as extreme temperatures or prolonged water deficit, triggering a transition from an endophytic to a pathogenic lifestyle and leading to severe symptoms in a broad range of woody hosts (Phillips et al. 2013; Yan et al. 2013; Paolinelli-Alfonso et al. 2016).

Despite their shared ecological characteristics, Botryosphaeriaceae species display considerable variation in host range and pathogenicity. Host associations analyses within Botryosphaeriaceae have revealed marked variation in host range among species (Silva-Valderrama et al. 2024). *Lasiodiplodia theobromae*, *Botryosphaeria dothidea*, and *Neofusicoccum parvum* are documented on hundreds of hosts, whereas many other species exhibit much narrower host ranges. Substantial variation in virulence has also been reported among Botryosphaeriaceae species. A study of 72 strains from nine Botryosphaeriaceae species associated with grapevine cankers demonstrated large differences in their ability to cause canker symptoms (Úrbez-Torres and Gubler 2009). Similar interspecific variation in pathogenicity was also observed among eight Botryosphaeriaceae species isolated from apple stems and fruits (Delgado-Cerrone et al. 2016). These ecological and pathogenic differences raise important questions about the genetic mechanisms underlying host range and virulence variation within Botryosphaeriaceae.

Recent advances in comparative genomics have begun to shed light on the genetic basis of these differences. These studies revealed prevalent gene family expansions in Botryosphaeriaceae species, suggesting that gene family innovations may contribute to their diverse infection strategies and host adaptations (Wang et al. 2018; Yan et al. 2018; Garcia et al. 2021; Nagel et al. 2021). Such functional innovations, including CAZymes, secondary metabolism enzyme clusters (SM), proteases, cytochrome P450 enzymes, transcription factors (TFs), and transporters, as well as secreted effectors that modulate host immunity, provide insights into fungal pathogenesis and virulence, including the ability to colonize and damage host tissues (Dou and Zhou 2012; Hartmann and Croll 2017; Rogers et al. 2022). Variation in the abundance and diversification of these gene families may therefore contribute to differences in virulence and infection strategies among Botryosphaeriaceae species.

Within Botryosphaeriaceae, the genera *Botryosphaeria*, *Neofusicoccum*, and *Lasiodiplodia* represent the most virulent genera associated with grapevine. Recent studies showed that these genera experienced extensive gene family expansions, particularly in secreted hydrolytic enzymes and secondary metabolic biosynthetic gene clusters (Nagel et al. 2021). Lineage-specific expansion of pathogenicity-related genes has also been reported in these taxa (Garcia et al. 2021). Although gene family expansions have received considerable attention, relatively fewer gene family contractions have been documented in Botryosphaeriaceae. However, studies in other pathogens suggest that gene losses can accompany shifts in growth form or pathogenicity and may represent an alternative mechanism of host adaptation (Hartmann and Croll 2017; Rogers et al. 2022; Zhu et al. 2023).

Thus, elucidating how gene family innovations, including both expansions and contractions, affect the pathogenicity and evolutionary adaptation of Botryosphaeriaceae remains an important direction for further investigation. This study aims to provide new insights into the relations between gene family innovations and host infection in Botryosphaeriaceae. Preliminary analyses indicated substantial genomic variation among strains, suggesting that a single genome may not adequately represent species-level diversity. To better capture this variation and provide a robust basis for comparative and evolutionary analyses, multiple genomes were generated using PacBio long-read sequencing (Fig. S1). Specifically, five genomes were newly sequenced from strains exhibiting pronounced differences in virulence during grapevine infection, including two *B. dothidea* strains, two *N. parvum* strains, and one *L. theobromae* strain. These five high-quality genomes, together with four previously published genomes and 47 publicly available fungal genomes from the RefSeq database, were utilized to identify gene family innovations and explore their potential roles in the evolution of pathogenicity in Botryosphaeriaceae (Goldfarb et al. 2024; Wang et al. 2024).

## Results

### Virulence Variation of Botryosphaeriaceae Strains

**Fig. 1.**
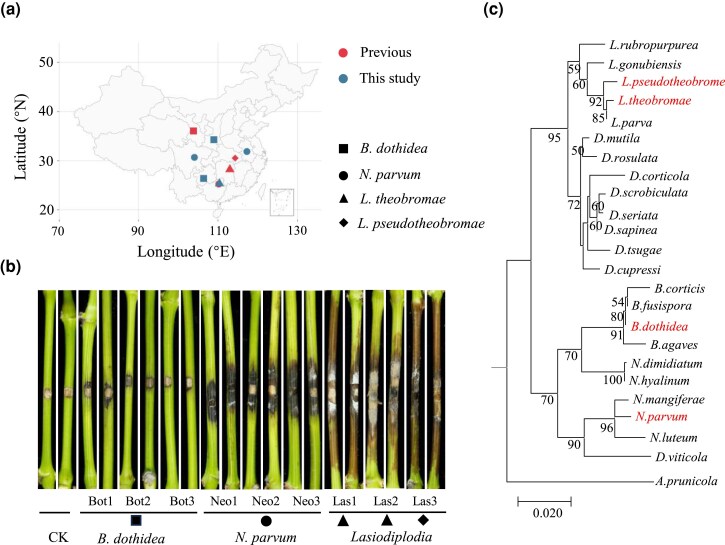
Geographic distribution, virulence comparison, and phylogeny of Botryosphaeriaceae strains used in this study. a) Geographic distribution of the nine Botryosphaeriaceae strains. Previous: indicates four strains whose genomes were sequenced in a previous study (Wang et al. 2024). This study: indicates five strains newly sequenced here. b) Lesion length comparison for the nine strains on 1-year-old shoots of grapevine cultivar “Summer Black” at 11 d postinoculation; statistical differences are shown in Fig. S2b. c) Maximum likelihood phylogeny of Botryosphaeriaceae inferred from concatenated ITS2 and LSU sequences using RAxML-NG. *Aplosporella prunicola* was used as the outgroup. Numbers at nodes indicate bootstrap support values. The four focal species in this study are *B. dothidea*, *N. parvum*, *L. theobromae*, and *L. pseudotheobromae*.

We first focused on nine Botryosphaeriaceae strains to examine virulence variation, including five newly sequenced strains and four strains whose genomes we previously sequenced (Wang et al. 2024). These strains were selected from our collection of grapevine-associated Botryosphaeriaceae isolates obtained from different grape-growing regions of China, with the aim of representing geographic diversity among the available isolates. The nine strains comprised three *B. dothidea* strains, three *N. parvum* strains, two *L. theobromae* strains, and one *Lasiodiplodia pseudotheobromae* strain, all isolated from grapevine stems (Fig. 1a, Fig. S2a).

Inoculation assays showed significant differences in virulence, as assessed by lesion length, among the three genera (ANOVA, *F* = 287.42, *P* < 2.2 × 10^−16^). *Lasiodiplodia* produced the longest lesions, whereas *B. dothidea* produced the shortest lesions. No significant intraspecific differences in lesion length were detected within *B. dothidea*, *Lasiodiplodia*, or *N. parvum* (Fig. 1b and Fig. S2b). These results indicate that *Lasiodiplodia* strains exhibit higher virulence than the other Botryosphaeriaceae taxa examined.

We then assessed whether phylogenetic relationships among these species could explain the observed differences in virulence. To elucidate the evolutionary relationships among these four species, a phylogenetic analysis was conducted based on a concatenated dataset of ITS2 and the ribosomal LSU gene sequences from these four species and 19 additional Botryosphaeriaceae species, with *Aplosporella prunicola* used as the outgroup. The analyses revealed that the 23 Botryosphaeriaceae species were divided into two lineages. The *B. dothidea* and *N. parvum* were clustered in the same lineage, while *L. theobromae* and *L. pseudotheobromae* were clustered in another lineage (Fig. 1c). These results indicate a general correlation between phylogenetic lineage and virulence patterns, motivating the sequencing of five additional strains to systematically examine the genomic basis of variation in pathogenic potential among Botryosphaeriaceae species.

### Assembly of Botryosphaeriaceae Genomes

To explore the genomic determinants of virulence variation in Botryosphaeriaceae, genome assemblies for the five newly sequenced strains were generated using methods similar to those in our previous study (Wang et al. 2024). Genome sequencing was conducted using a combination of PacBio Sequel and Illumina technologies, generating an average of 5.3 and 4.2 Gbp of sequence data per genome, respectively (∼100× coverage; Tables S1 to S3). The BUSCO scores were >99% for all assemblies, and >99.5% of the Illumina reads could be mapped to the assemblies, suggesting a high degree of completeness for all five genomes (Tables S4 to S5).

The newly generated PacBio assemblies comprised between 24 and 178 scaffolds, with genome sizes ranging from 44 to 47 Mbp. The predicted gene number for these genomes ranged from 12,833 to 13,527 (Table S6). To validate the quality of our genome assemblies, we compared a combined set of nine newly sequenced strains (five from this study and four from our prior work) with 11 publicly available Botryosphaeriaceae genomes (five *B. dothidea*, three *N. parvum*, and three *L. theobromae*) (Table S7). The assemblies of our nine strains consistently exhibited longer genome lengths and higher N50 values than those of the previously published genomes (Fig. S3). Furthermore, genome sizes and gene numbers in these nine strains were significantly greater than those of the 504 Ascomycota genomes available in the RefSeq database (Wilcoxon rank-sum test, *W* = 1250, *P* = 0.001 for genome size, and *W* = 1211, *P* = 0.003 for gene number; Fig. S4a and b). This indicates that genome expansion is more pronounced in Botryosphaeriaceae relative to other Ascomycota species. The transposable element (TE) content in these nine genomes ranged from 5.04% to 10%, similar to TE contents reported for other Ascomycota genomes (*W* = 1119, *P* = 0.07), implying a minimal contribution of TEs to genome expansion in Botryosphaeriaceae (Fig. S4c).

### Genome Collinearity Between Nine Botryosphaeriaceae Strains

To assess whether virulence differences among Botryosphaeriaceae strains are associated with variation in genome structural conservation, we analyzed genomic collinearity. Orthologous genes identified across the genomes were used to assess genome collinearity using the MCScan method (Wang et al. 2012).

Genome collinearity varied across Botryosphaeriaceae species. Within each species, collinearity was generally high, exceeding 92% (Fig. S5). Within *Lasiodiplodia*, *L. theobromae* and *L. pseudotheobromae* showed approximately 89% collinearity, indicating that congeneric species largely preserve genome structure (Fig. S5e). However, comparisons across genera revealed lower collinearity, with less than 70% of the genomes conserved between species from different genera, reflecting substantial sequence and structural divergence following genus-level separation.

### Widespread Lineage-Specific Gene Family Innovations in Botryosphaeriaceae

Given the observed variation in genome collinearity, we next investigated whether these structural differences are associated with lineage-specific gene family innovations. To this end, we compared gene family sizes across nine Botryosphaeriaceae genomes and 47 representative Ascomycota genomes (Table S8) to identify innovations that may underlie differences in host interaction, virulence, and ecological adaptation.

**Fig. 2.**
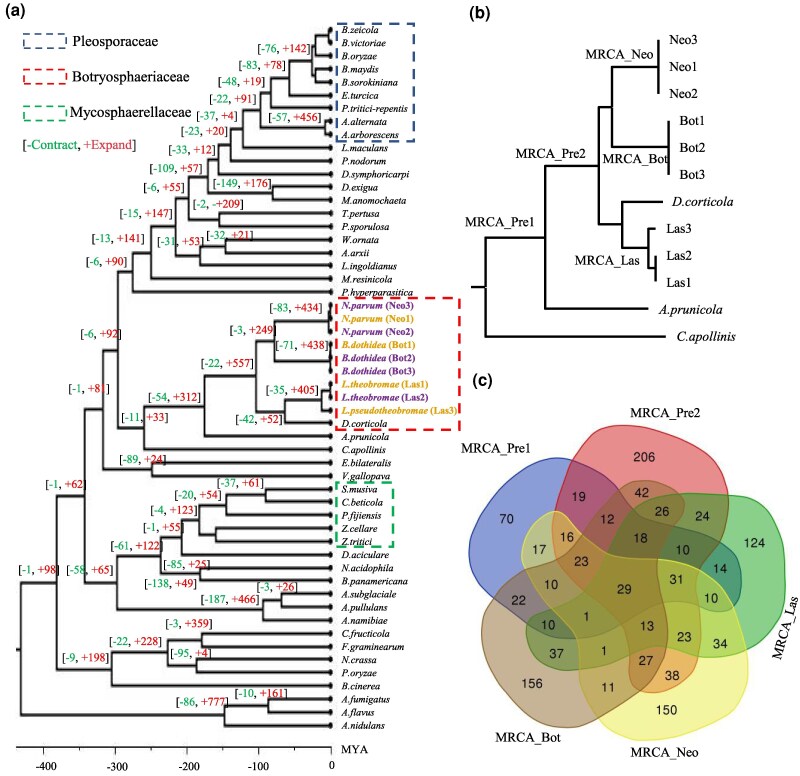
Gene family innovations in Botryosphaeriaceae. a) Gene family expansion and contraction inferred from a time-calibrated phylogeny of 54 fungal strains representing 49 species. The phylogeny was reconstructed based on concatenated single-copy genes. Numbers in brackets indicate the numbers of gene family gains and losses at each node. Dashed boxes indicate species from three families within Dothideomycetes: Pleosporaceae, Botryosphaeriaceae, and Mycosphaerellaceae. The nine Botryosphaeriaceae strains analyzed in this study are labeled with strain names in parentheses. b) Schematic illustration of the positions of different MRCA nodes within the time-calibrated phylogeny of Botryosphaeriales. c) Venn diagram showing expanded gene families at five ancestral nodes in Botryosphaeriales. Numbers indicate the number of expanded gene families.

Using OrthoFinder, we identified 24,807 orthologous gene families across 56 genomes, with 11,814 families present in at least one Botryosphaeriaceae strain. Comparative analyses revealed extensive gene family expansions at multiple evolutionary nodes within Botryosphaeriaceae, especially in the most recent common ancestors (MRCAs) of *Botryosphaeria*, *Neofusicoccum*, *Diplodia*, and *Lasiodiplodia* (557 expansions), which is substantially higher than in Mycosphaerellaceae (123 families) or Pleosporaceae (91 families) (Fig. 2a). These large-scale expansions are unlikely to be an artifact of genome assembly quality (Fig. S4d). More ancient expansions were also identified in the MRCA of these four Botryosphaeriaceae genera and *A. prunicola* within the Aplosporellaceae of Botryosphaeriales. These two events, hereafter referred to as MRCA_Pre2 and MRCA_Pre1, respectively, were deeply rooted within Botryosphaeriales prior to the divergence of Botryosphaeriaceae into genera (Fig. 2a and b).

Lineage-specific expansions were further observed in the MRCA of *Botryosphaeria* and *Neofusicoccum* and the MRCA of *Diplodia* and *Lasiodiplodia*, as well as in the MRCA of *B. dothidea* (MRCA_Bot), *N. parvum* (MRCA_Neo), and *Lasiodiplodia* strains (MRCA_Las). Among MRCA_Las, MRCA_Neo, and MRCA_Bot, over 30% of the expanded families were unique to each lineage, highlighting the genus-specific nature of these expansions (Fig. 2c).

Functional enrichment analysis of the expanded gene families revealed significant enrichment of GO terms associated with pathogenicity-related processes, including transmembrane transport, carbohydrate metabolism, and oxidation–reduction. Mapping these enriched functions onto the phylogeny highlighted lineage-specific patterns, with oxidation–reduction and transport-related expansions particularly evident in the MRCA_Bot, MRCA_Neo, and MRCA_Las, suggesting that redox-associated adaptations may have contributed to differences in host interaction strategies.

Although contractions were less numerous than expansions, they were also observed at multiple nodes (Fig. 2a). Interestingly, oxidation–reduction-related GO terms were significantly enriched among contracted families in MRCA_Las, MRCA_Neo, and MRCA_Bot (Fig. S6b). This suggests that both gene family expansions and contractions may have contributed to functional diversification, including processes potentially relevant to pathogenicity, within Botryosphaeriaceae.

### Lineage-Specific Gene Family Innovations Contributed to Host Infection in Botryosphaeriaceae

To investigate the role of gene family innovations during infection, publicly available dual RNA-seq data (GSE190625) from *Vitis vinifera* cv. “Summer Black” stems infected with Las1 for 24 h were analyzed to identify infection-related genes and families. Fungal reads were extracted and compared with the Las1 transcriptome from cultures on potato dextrose agar (PDA) (Table S9). Las1 contains 13,095 predicted genes, which were assigned to 9,316 gene families. Among these genes, 7,516 were expressed in at least one condition (FPKM >1). Differential expression analysis using the limma package identified 2,473 differentially expressed genes during infection compared with PDA-grown cultures, including 1,090 upregulated genes and 1,383 downregulated genes (adjusted *P* < 0.05 and fold change >1.5). At the gene family level, 6,638 of the 9,316 Las1 gene families contained at least one expressed gene, with 950 families showing induced expression and 1,363 showing repressed expression under infection.

**Fig. 3.**
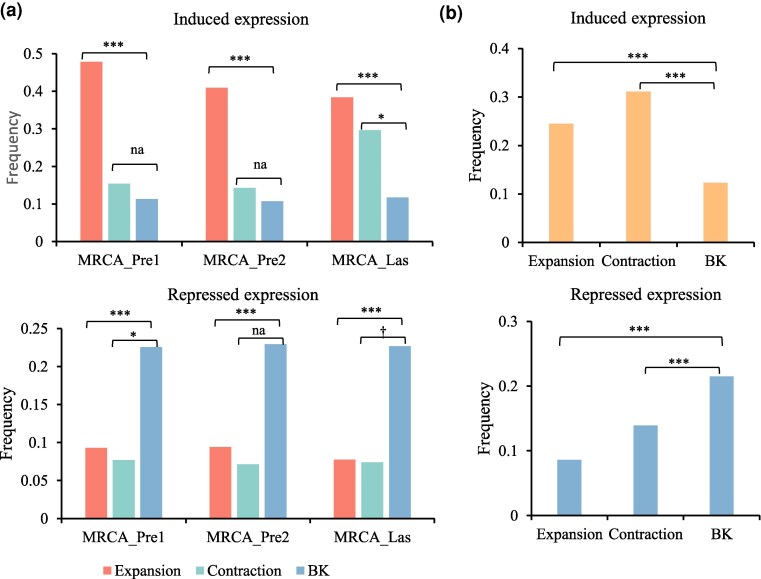
Transcriptome analysis of gene family expansion and contraction in Botryosphaeriaceae. a) Frequency of induced and repressed genes in expanded (expansion), contracted (contraction), and stable (BK) gene families after inoculation of grapevine shoots with Las1 for 24 h. Induced and repressed genes were identified by comparing fungal transcripts from infected grapevine shoots (24 h postinoculation) with those from Las1 grown on PDA (*P* < 0.05, fold change ≥1.5). For each ancestral node, differences in the proportions of induced or repressed genes in Expansion and Contraction relative to BK were assessed using Fisher's exact test. (b) Frequency of induced and repressed genes in *Lasiodiplodia* gene families with either higher (Expansion) or lower (Contraction) gene copy numbers compared with the six strains of *B. dothidea* and *N. parvum*. In this analysis, BK represents all other gene families in *Lasiodiplodia* excluding Expansion and Contraction. Differences in the proportions of induced or repressed genes between Expansion or Contraction and BK were evaluated using the chi-square test. ***P < 0.001; *P < 0.05; †*P* < 0.1; na: *P* > 0.1.

To assess the contribution of gene family innovation to infection, we focused on the three key evolutionary stages, MRCA_Pre1, MRCA_Pre2, and MRCA_Las, and categorized gene families into expanded, contracted, or stable (“BK”). A gene family was defined as induced if at least one member was upregulated after inoculation and as repressed if at least one member was downregulated. The frequency of induced gene families in the expansion groups was significantly higher than in the BK group (Fisher's exact test, all *OR* > 4.7 and *P* < 2.2 × 10^−16^; Fig. 3a). Interestingly, the contraction group of MRCA_Las also showed an elevated proportion of induced families compared with the BK group, a pattern not observed in MRCA_Pre1 and MRCA_Pre2. Conversely, both expansion and contraction groups tended to exhibit a lower frequency of repressed families compared with BK. This reduction was statistically significant for MRCA_Pre1 (Fisher's exact test, *OR* = 0.28, *P* = 0.03) and marginally significant for the contraction groups of MRCA_Las (*OR* = 0.27, *P* = 0.06). These results suggest that both expansion and contraction of gene families contribute to transcriptional response during Las1 infection.

Comparing the three *Lasiodiplodia* strains with six *B. dothidea* and *N. parvum* strains, we identified 208 expanded and 215 contracted gene families specific to *Lasiodiplodia*. These lineage-specific families were enriched in functions potentially associated with host infection, including oxidation–reduction, zinc ion binding, hydrolase and metallopeptidase activities, and transmembrane transport (Table S10). Consistent with the MRCA-level patterns, these families contained a higher proportion of induced genes but fewer repressed genes compared with families showing no copy number variation (Fig. 3b). Collectively, these results demonstrate that lineage-specific gene family expansions and contractions contributed to the infection process of *Las1*.

### Lineage-Specific Innovations of the Pathogenicity Genes in Botryosphaeriaceae

To investigate lineage-specific innovations of pathogenicity genes in Botryosphaeriaceae, we compared gene families associated with host infection and pathogenicity across 56 fungal genomes. The nine Botryosphaeriaceae genomes encoded abundant CAZymes (Fig. S7) and showed a significantly expanded repertoire of SM genes, protease genes, P450 genes, and transporter genes relative to other fungi (Welch's *t*-test, all *t* > 5.52 and *P* < 1.52 × 10^−6^). Comparisons within Botryosphaeriaceae further revealed genus-level differences in these functional categories, suggesting divergent host infection strategies among *Botryosphaeria*, *Neofusicoccum*, and *Lasiodiplodia* (Fig. S7).

Because *Lasiodiplodia* strains caused longer lesions than *B. dothidea* and *N. parvum*, we next focused on CAZyme families to identify candidate genetic factors associated with these virulence differences. Seven CAZyme families, including AA11, CE12, GH106, GH11, GH43, GH63, and PL1, were consistently expanded in *Lasiodiplodia* relative to *B. dothidea* and *N. parvum*, whereas 21 CAZyme families were contracted (Table 1). These contrasting patterns suggest that lineage-specific remodeling of CAZyme repertoires may contribute to differences in host infection strategies among Botryosphaeriaceae species. Among the expanded CAZyme families, GH11 was selected for further analysis because GH11 xylanases degrade xylan, a major hemicellulosic component of plant cell walls, and have been implicated in fungal pathogenicity (Noda et al. 2010; Rafiei et al. 2021).

**Table 1:** CAZyme families showing copy number differences in *Lasiodiplodia* compared with *B. dothidea* and *N. parvum*

	Botryosphaeria			Neofusicoccum			Lasiodiplodia		
	Bot1	Bot2	Bot3	Neo1	Neo2	Neo3	Las1	Las2	Las3
AA11	2	2	2	2	2	2	4	4	5
AA4	4	4	4	6	6	6	3	3	3
AA8	4	4	4	4	4	4	3	3	3
CBM1	11	12	11	11	11	10	7	7	7
CBM32	2	2	2	1	1	1	0	0	0
CBM63	5	3	3	3	3	3	2	2	2
CE12	4	4	4	4	4	4	5	5	5
CE5	11	11	11	9	9	9	6	6	6
GH1	8	6	6	5	5	5	4	4	4
GH10	6	6	6	5	6	6	4	4	4
GH106	1	2	2	2	2	2	4	4	3
GH11	1	1	1	1	1	1	2	2	2
GH125	3	3	3	3	3	3	2	2	2
GH127	1	1	1	1	1	1	0	0	0
GH18	12	13	12	11	10	10	9	8	9
GH29	3	5	3	3	3	3	2	2	2
GH30	1	1	1	1	1	1	0	0	0
GH31	10	10	10	10	10	10	8	8	8
GH33	1	1	1	1	1	1	0	0	0
GH39	2	2	2	2	2	2	0	0	0
GH43	16	16	16	15	15	15	18	18	20
GH63	1	1	1	1	1	1	2	2	2
GH65	1	1	1	1	1	1	0	0	0
GH92	6	6	6	7	6	6	5	5	4
GH93	2	2	2	3	3	3	1	1	1
GT2	18	17	16	15	16	15	14	14	14
GT48	2	2	2	2	2	2	1	1	1
PL1	8	8	8	8	8	7	10	10	10

Notes: Numbers indicate gene copy numbers in each CAZyme family. Family names in red denote higher copy numbers in *Lasiodiplodia*, whereas those in green denote lower copy numbers compared with *B. dothidea* and *N. parvum*.

### Lineage-Specific Expansion of the GH11 Family Contributes to *L. theobromae* Virulence

**Fig. 4.**
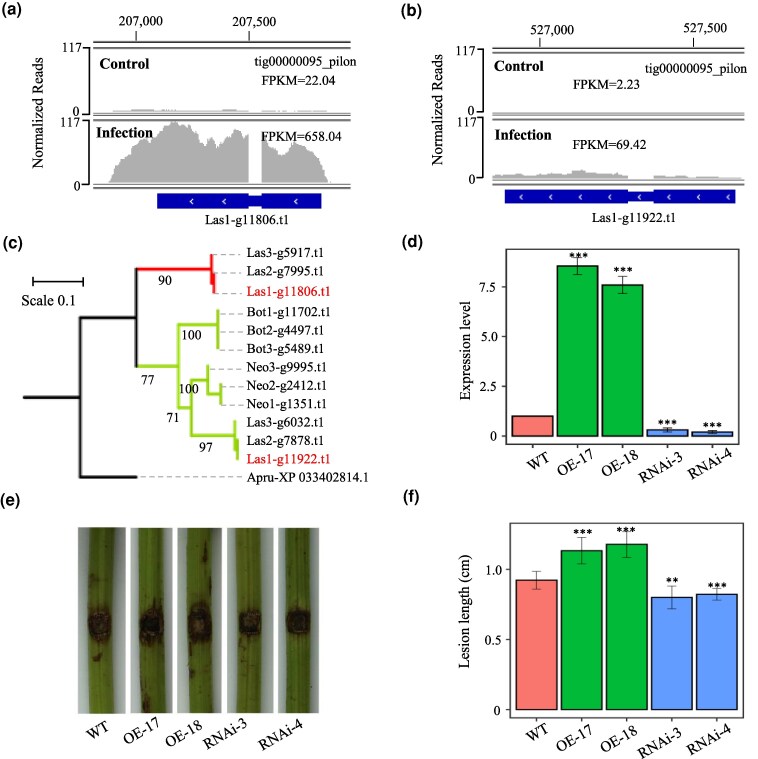
Lineage-specific expansion of GH11 contributes to host virulence in Las1. a and b) Expression levels of the two GH11 copies, g11806.t1 (a) and g11922.t1 (b), in Las1 under in vitro conditions (Control) and in vivo during infection of grapevine shoots at 24 hpi (Infection). c) Maximum likelihood phylogenetic tree of GH11 homologs identified in the nine genomes. Numbers at nodes indicate bootstrap support values based on 1,000 replicates. d) qRT-PCR analysis of g11806.t1 expression in overexpression (OE) and RNAi-silenced transformants compared with the wild-type strain (WT). Relative expression levels were determined by qRT-PCR (three biological replicates) and normalized to WT. e) Representative lesions on grapevine shoots 48 h after inoculation with OE and RNAi transformants of g11806.t1. f) Statistical analysis of lesion lengths shown in (e). Each strain included 10 biological replicates; error bars represent the standard error. Statistical significance was determined by a two-tailed Welch's *t*-test relative to WT. **P < 0.01, ***P < 0.001.

Two GH11 members were present in *Lasiodiplodia*, while only one was found in *B. dothidea* and *N. parvum*. Transcriptomic analysis showed that both GH11 genes in Las1, g11806.t1 and g11922.t1, were upregulated after 24 h of inoculation on grapevine branches. The fold inductions of both genes were within a comparable order of magnitude, indicating that both contribute to the early infection response (Fig. 4a and b). Notably, g11806.t1 exhibited higher absolute expression (FPKM = 658.04) than g11922.t1 (FPKM = 69.42). Phylogenetic analyses revealed that g11806.t1 is more distantly related to the GH11 homologs in *B. dothidea* and *N. parvum* than g11922.t1 (Fig. 4c), suggesting that g11806.t1 may play a unique and important role in *Lasiodiplodia* virulence.

To investigate the role of g11806.t1 in Las1 virulence, the gene was overexpressed and silenced in vivo via PEG-mediated protoplast transformation. Transformants were screened for neomycin resistance and validated by qRT-PCR (Fig. 4d). Pathogenicity assays showed that two overexpression transformants caused longer lesions on grapevine branches than the wild type (Welch's *t*-test, *t* = 5.27, *P* = 1.80 × 10^−4^ for OE-17 and *t* = 6.51, *P* = 2.00 × 10^−5^ for OE-18), whereas two RNAi transformants produced shorter lesions (*t* = −3.35, *P* = 1.2 × 10^−3^ for RNAi-3 and *t* = −3.75, *P* = 8.00 × 10^−4^ for RNAi-4; Fig. 4e and f), indicating that g11806.t1 contributes to Las1 virulence.

GH11 xylanases in phytopathogenic fungi degrade xylan in plant cell walls to facilitate host infection. To assess the role of g11806.t1 in carbon source utilization, Las1 and its transformants were cultured on media containing cellulose, CMC-Na, xylan, glucose, or xylose as the sole carbon sources. Overexpression transformants grew significantly faster than the wild type on xylan (Welch's *t*-test, *t* = 5.47, *P* = 0.002; Fig. S8), while RNAi transformants showed slower growth (*t* = −2.22, *P* = 0.078). No growth differences were observed on other carbon sources. These results suggest that g11806.t1 enhances the degradation of xylan in plant cell walls.

## Discussion

In this study, we generated high-quality genome assemblies for five strains representing three genera within the Botryosphaeriaceae and conducted a comparative genomic analysis to investigate how gene family innovations shape the evolution of pathogenicity. Building upon our previous work, which focused on ancestral genome expansion and horizontal gene transfer during the early evolution of the Botryosphaeriaceae (Wang et al. 2024), the present study shifts the perspective toward lineage-specific innovations that occurred after genus divergence and examines their direct connections to pathogenic diversification and virulence evolution. Our findings reveal multiple rounds of gene family expansion and contraction across the evolutionary history of this fungal family, including both ancient events that predate genus divergence and more recent lineage-specific changes associated with differences in virulence among species.

Gene family expansion and contraction are widely recognized as powerful evolutionary forces driving adaptation across diverse fungal lineages. Expansions typically generate novel gene copies that diversify in function, while contractions streamline genomes and promote specialization (Rogers et al. 2022; Steindorff et al. 2024; Feng et al. 2025). Comparative studies have shown that gene family turnover underpins key ecological transitions in fungi, including shifts from saprotrophy to parasitism or symbiosis (Merényi et al. 2023; Steindorff et al. 2024; Fijarczyk et al. 2025). In plant pathogens, expansions in CAZymes, transporters, and secondary metabolism genes are strongly associated with host specialization and virulence diversity (Morales-Cruz et al. 2015; Baroncelli et al. 2016, 2024; Sipos et al. 2017). Our results are consistent with these broader patterns: ancient expansions in transporter families likely supported the transition from saprotrophy to pathogenicity in early Botryosphaeriaceae (Wang et al. 2024), whereas more recent expansions in ion-binding and oxidoreductase genes contributed to lineage-specific adaptations such as iron homeostasis and stress tolerance, key factors influencing virulence (Perlin et al. 2014; Liu et al. 2021).

We further identified lineage-specific expansion of GH11 xylanases in *Lasiodiplodia*, which encode enzymes known to degrade plant cell wall xylan and promote necrosis (Noda et al. 2010; Rafiei et al. 2021; Wang et al. 2024). The presence of two GH11 in *Lasiodiplodia*, compared to one in *B. dothidea* and *N. parvum*, and their strong induction during infection suggest that duplication of pathogenicity-related genes may have contributed to the enhancement of virulence in this genus.

While expansion is often highlighted, gene loss is also a pervasive driver of adaptation (Sharma et al. 2018; Helsen et al. 2020). Gene family contractions can facilitate specialization by removing redundant or energetically costly functions (Steenwyk et al. 2019; Helsen et al. 2020; Merényi et al. 2023). This process has been documented across diverse fungal lineages, where the reduction of gene content often coincides with ecological transitions and niche specialization. For example, *Calonectria* phytopathogens show rapid contraction of pathogenesis-related genes, whereas saprobic species retain broader metabolic repertoires (Rogers et al. 2022). Our analyses revealed that contractions in *Lasiodiplodia*, *B. dothidea*, and *N. parvum* were enriched in oxidation–reduction processes, and that genes from these contracted families were frequently induced during host infection (Fig. 3). These results indicate that gene loss, rather than being purely degenerative, can be selectively advantageous by refining infection strategies. Such adaptive gene losses have also been reported in fungi undergoing ecological specialization or adaptation to extreme environments (Merényi et al. 2023; Fijarczyk et al. 2025).

Overall, these results fit into a broader evolutionary framework in which both gene family expansion and contraction have repeatedly shaped fungal adaptation and pathogenic diversification. From an evolutionary perspective, such patterns may reflect shifts in selective constraints associated with gene family turnover: gene family expansion can relax purifying selection on duplicated copies and facilitate functional diversification, whereas gene family contraction may occur when certain ancestral functions become dispensable during ecological specialization. Acting in concert, expansions introduce new functions that enable ecological exploration, whereas contractions refine existing repertoires and streamline metabolism for specialization. Such complementary processes have driven major evolutionary transitions across fungi, from extensive gene loss in obligate parasites like powdery mildews and *Pneumocystis* (Spanu et al. 2010; Steenwyk et al. 2019) to lineage-specific expansions of CAZyme and secondary metabolism genes in *Fusarium* and *Colletotrichum* (Niehaus et al. 2016; Meng and Tian 2023; Baroncelli et al. 2024). These patterns underscore that the evolutionary success of fungi, including the Botryosphaeriaceae, depends on a dynamic balance between gene gain and loss that enables continual adaptation to changing ecological niches.

Taken together, our findings demonstrate that both gene family expansions and contractions contribute to the evolution of pathogenicity within the Botryosphaeriaceae. Through lineage-specific gene gain or loss, pathogens can modify their virulence and ecological strategies, leading to diversification in pathogenic potential during evolution.

## Materials and Methods

### Strains and Pathogenicity Analysis

The five strains evaluated in this study were collected from *V. vinifera* plants grown in different provinces of China and deposited at the Beijing Academy of Agricultural and Forestry Sciences, Beijing, China.

Healthy, 1-year-old green shoots of the grapevine cultivar “Summer Black,” with a diameter of 4 to 6 mm, were collected from the field for pathogenicity testing. Shoots were pruned to approximately 30 cm in length and surface-sterilized using 75% ethanol wipes. To inoculate the Botryosphaeriaceae isolates, wounds of 4 mm diameter were created on each shoot using a cork borer, and a 4 mm agar plug containing the fungal isolate was then placed into each wound. The inoculation site was then sealed with Parafilm (Bemis, Oshkosh, WI, United States). Nine shoots were used as biological replicates for each isolate, while sterile PDA plugs served as controls. Inoculated shoots were planted in sterilized, moist soils in small nursery pots and incubated in a controlled chamber at 25 °C under an 8 h light and 16 h dark cycle. The relative humidity was maintained at 80% for the first 2 d of postinoculation. Lesion lengths were measured and photographed after 5 d in three independent experiments.

### DNA Extraction, Genome Sequencing, and Assembly

For long-read sequencing, total genomic DNA from the five strains was extracted using the CTAB method. DNA libraries were subsequently prepared with the Pacific Biosciences Express Template Prep Kit and sequenced on the PacBio Sequel platform (Pacific Biosciences, CA, United States). Each strain yielded 4.04 to 7.11 Gbp of raw data, which were quality-filtered using the SMRTlink software (version 5.0, https://www.pacb.com/support/software-downloads). Concurrently, short-read sequencing was performed on the Illumina X ten platform using 150 bp paired-end reads with an average fragment size of 500 bp. Raw reads were first assessed for quality using FastQC (Wingett and Andrews 2018), and then low-quality reads were removed with Trimmomatic using default parameters (version 0.30) (Bolger et al. 2014).

PacBio reads were corrected using the Canu software (Koren et al. 2017), then three different assemblers were used for the initial genome assemblies independently, including Canu (version 2.1), WTDBG2 (Ruan and Li 2020), and MASURCA (version 3.3.8) (Zimin et al. 2013). All assemblies were performed under default parameters. The initial genome was then polished with Pilon using the Illumina short reads. We merged the draft Canu assemblies with the MASURCA and WTDBG2 assemblies separately using the Quickmerge program (version 0.3) (Solares et al. 2018), and further polished using Pilon (version 1.23) (Walker et al. 2014).

Each assembly was first assessed for completeness using BUSCO v3.0.2 (fungi odb9 dataset, total of 290 BUSCOs) (Manni et al. 2021), followed by quality evaluation via mapping Illumina reads back to the assemblies with BWA v0.7.17 (version 0.7.17) to further evaluate assembly quality under default parameters (Li and Durbin 2010). The highest-quality genomes were subsequently selected based on their BUSCO scores and read mapping frequency.

### Genome Annotation and Gene Prediction

For each genome, a de novo repeat library was constructed using RepeatModeler (version 1.0.11) (Saha et al. 2008). Repeat sequences were subsequently annotated by RepeatMasker (version 4.0.9, http://www.repeatmasker.org) under default parameters. Protein genes were predicted using BRAKER (version 2) with the model of proteins of any evolutionary distance using the OrthoDB fungal protein database (version 10) (Brůna et al. 2021). To evaluate the quality of gene prediction, the predicted proteomes for each genome were assessed using BUSCO v3.0.2 with the fungi_odb9 dataset (Manni et al. 2021).

### Gene Functional Annotation

We predicted carbohydrate-active enzymes (CAZymes) using the dbCAN2 system (version 2) (Zhang et al. 2018), which integrates predictions from HMMER, DIAMOND, and Hotpep. Secondary metabolite biosynthesis gene clusters and cytochrome P450s were identified with antiSMASH. Two programs, antiSMASH (version 5.2.0) (Blin et al. 2021) and InterProScan (version 5.52-86.0) (Jones et al. 2014), were used to predict putative secondary metabolite biosynthesis clusters and cytochrome P450s. Proteins with signal peptides predicted by SignalP (version 4.1) (Almagro Armenteros et al. 2019) and TargetP (version 2) (Emanuelsson et al. 2007), along with the lack of transmembrane domains predicted by TMHMM (Krogh et al. 2001), were regarded as predicted secreted proteins. EffectorP (version 2) was used to predict candidate effectors (Sperschneider et al. 2016). All the above tools were run with their default parameters. MEROPS database (release 12.3) was used to identify putative proteases with a BLASTP approach and an *E*-value cut-off of 1e^−5^ (Rawlings et al. 2018).

For each genome, protein domains and Gene Ontology (GO) annotations were predicted using InterProScan (version 5.52-86.0) (Jones et al. 2014) with default parameters. GO terms were assigned to a gene family only when presented in at least three of the nine strains. GO enrichment analysis was performed using the hypergeometric test, and GO terms with adjusted *P* < 0.05 were considered statistically significant. TFs were identified based on InterProScan IPR terms corresponding to entries in the Fungal Transcription Factor database (Park et al. 2008). PFAM annotations obtained from InterProScan were also mapped to the Transporter Classification Database (TCDB) to identify putative transporter proteins (Saier et al. 2021).

### Genomic Data Collection

Genome assemblies and annotated protein sequences for 47 Ascomycota fungi were retrieved from the RefSeq database, including *Saccharomyces cerevisiae*, *Schizosaccharomyces pombe*, and 45 additional species within the Pezizomycotina subphylum. Among the Pezizomycotina species, 37 belonged to the class Dothideomycetes, including *Aaosphaeria arxii*, *Alternaria alternata*, and *Zymoseptoria tritici*; three belonged to the Eurotiomycetes, including *Aspergillus flavus*, *Aspergillus fumigatus*, and *Aspergillus nidulans*; one belonged to the Leotiomycetes, *Botrytis cinerea*; and four belonged to the Sordariomycetes, including *Fusarium graminearum*, *Neurospora crassa*, *Pyricularia oryzae*, and *Colletotrichum fructicola* (Table S8).

### Phylogenomic Analyses

ITS and LSU sequences from 23 species representing six genera of the Botryosphaeriaceae, along with *A. prunicola*, were retrieved from GenBank (Table S7). Each locus was aligned separately using MAFFT (version 7.313) with default parameters (Katoh and Standley 2013). Spurious sequences and poorly aligned regions were removed from the multiple sequence alignments using trimAl (version 1.2) with the parameter -automated1 (Capella-Gutiérrez et al. 2009), and the resulting alignments were concatenated. Maximum likelihood phylogenetic inference was performed using RAxML-NG with the parameters --all, --model TIM3+I+G4, and --bs-trees 1,000 (Kozlov et al. 2019). The phylogenetic trees were rooted using *A. prunicola* sequences.

Single-copy core orthologous genes were identified across 56 Ascomycota species (or strains) using BUSCO v3.0.2 with the fungi_odb9 dataset (Manni et al. 2021). Nucleotide sequences of each gene were aligned using MAFFT, and poorly aligned regions or spurious sequences were removed with trimAl. The resulting alignments were concatenated, and a species tree was constructed using RAxML-NG under the GTR+I+G4 substitution model with 100 bootstrap replicates.

### Divergence Time Estimation

Divergence times among fungal species were estimated using the Markov chain Monte Carlo (MCMC) method implemented in MCMCTREE within PAML (version 4.9i) (Yang 1997). Fossil calibration points were obtained from the Time Tree database (http://www.timetree.org), including divergence times between *A. fumigatus* and *A. flavus* (0.55 to 0.94 Mya), *N. crassa* and *P. oryzae* (1.65 to 2.64 Mya), and *N. acidophila* and *B. panamericana* (1.27 to 2.27 Mya). Independent clock rates (clock = 2) and a maximum root age constraint (RootAge <500 Mya) were specified in the control file. MCMCTREE analyses were performed with the following parameters: burnin = 20,000, sampfreq = 100, and nsample = 100,000. Two independent MCMC runs were conducted to verify convergence.

### Gene Family Expansion and Contraction

For each of the 56 genomes, protein sequences without premature stop codons and longer than 50 amino acids were retained for gene family analysis. Proteins were clustered using the all-to-all DIAMOND method with an *E*-value threshold of 1 × 10^−5^ in OrthoFinder (Emms and Kelly 2019), resulting in 24,808 gene families. Gene family size evolution was analyzed using CAFÉ (version 5.0) (De Bie et al. 2006), which models gene gain and loss through a stochastic birth–death process. Because CAFÉ can yield biased estimates when gene families lack data for the most recent common ancestor (MRCA) in the phylogeny, *S. cerevisiae* and *S. pombe* were excluded from the analysis to minimize data loss. The dated species tree generated from the MCMCTREE analysis in PAML was used as the input phylogeny for evaluating gene family evolution.

### Transcriptome Analysis

Transcriptome data for Las1 inoculated onto detached *V. vinifera* cv. “Summer Black” branches for 24 h were downloaded from the GEO database (GSE190625). Transcriptome data from fungal mycelia grown on PDA plates for 6 h were used as the un-inoculated control. RNA-Seq reads were mapped to the Las1 genome using HISAT2 with default parameters (Kim et al. 2019). Differentially expressed genes were identified using the limma package (v.3.0.8) in R/Bioconductor, which fits a linear model to the expression data and applies an empirical Bayes method to moderate gene-wise variance. Genes with an adjusted *P* < 0.05 and a fold change >1.5 were considered significantly differentially expressed (Ritchie et al. 2015).

### Overexpression and RNAi Transformants of g11806.t1 in Las1 Used in Pathogenicity Analyses

The open reading frame of g11806.t1 was amplified using the primer pairs OE-f/OE-r (Table S11) and subcloned into the modified pKSNTP vector under the control of the PtrpC promoter. The resulting construct (pKSNTP: g11806.t1) was introduced into Las1 protoplasts via PEG-mediated transformation (Thilini Chethana et al. 2020). Transformants were screened for neomycin resistance and confirmed by qRT-PCR analyses. Two overexpressed transformants, OE-17 and OE-18, were selected for subsequent pathogenicity assays on detached shoots of *V. vinifera* cv. “Summer Black.”

Green grapevine shoots were inoculated with 5-mm mycelium plugs cut with a cork borer and incubated underalternating dark/light cycles in a growth chamber under the same humidity and temperature conditions described above. Lesion lengths were measured at 48 h postinoculation (hpi). Ten biological replicates were used for each g11806.t1 overexpression transformants.

For RNA interference (RNAi) assays, a sense fragment of g11806.t1 was amplified using primers RNAi-Sf/RNAi-Sr (Table S11), and an antisense fragment was amplified with the primers RNAi-ASf/RNAi-ASr (Table S11). Both fragments were sequentially ligated into the pRTN vector to generate the RNAi construct (pRTN: g11806.t1), which was introduced into Las1 protoplasts using the same PEG-mediated transformation protocol. Pathogenicity assays for RNAi transformants were performed as described for the overexpression transformants.

For carbon source usage analysis, Czapek-Dox medium was prepared with the following composition (g/L): K_2_HPO_4_, 1.0; MgSO_4_.7H_2_O, 0.5; KCl, 0.5; FeSO_4_, 0.01; NaNO_3_, 2.5; and agar 15. Las1 and its transformants were cultured on basal medium or medium supplemented with cellulose, CMC-Na, xylan, glucose, and xylose as the sole carbon source (20 g/L) at 25 °C for 72 h. Growth rates were determined from colony diameters on media containing different carbon sources relative to those on basal medium, with four biological replicates per treatment.

## Supplementary Material

evag178_Supplementary_Data

## Data Availability

The raw genome sequence reads generated in this study are available in the NCBI Sequence Read Archive under BioProject accession PRJNA785365. The corresponding whole-genome shotgun assemblies are available in the DDBJ/ENA/GenBank databases under accession numbers JAJQKF000000000 (Bot2), JAJQKE000000000 (Bot3), JAJQKC000000000 (Neo2), JAJQKB000000000 (Neo3), and JAJQJZ000000000 (Las2). The RNA-seq data analyzed in this study are available in the NCBI Gene Expression Omnibus under accession number GSE190625. All other data supporting the findings of this study are available within the article and its online supplementary material.
