# Correction to: Lineage-Specific Gene Family Innovations Underpin the Pathogenicity of Woody-Plant Pathogens in Botryosphaeriaceae

**DOI:** 10.1093/gbe/evag205

**Published:** 2026-08-17

This is a correction to: Xuncheng Wang, Junbo Peng, Linna Wu, Xinghong Li, Wei Zhang, Jiye Yan, Lineage-Specific Gene Family Innovations Underpin the Pathogenicity of Woody-Plant Pathogens in Botryosphaeriaceae, *Genome Biology and Evolution*, Volume 18, Issue 7, July 2026, evag178, https://doi.org/10.1093/gbe/evag178

In the originally published version of the paper, the sampling location of one strain (Bot3) was incorrectly displayed in published figures. Specifically, the sampling location was shown as “Guangxi”, whereas the correct sampling location is “Guizhou”. This information is corrected in panel a of Figure 1 which should now read:

**Figure:**
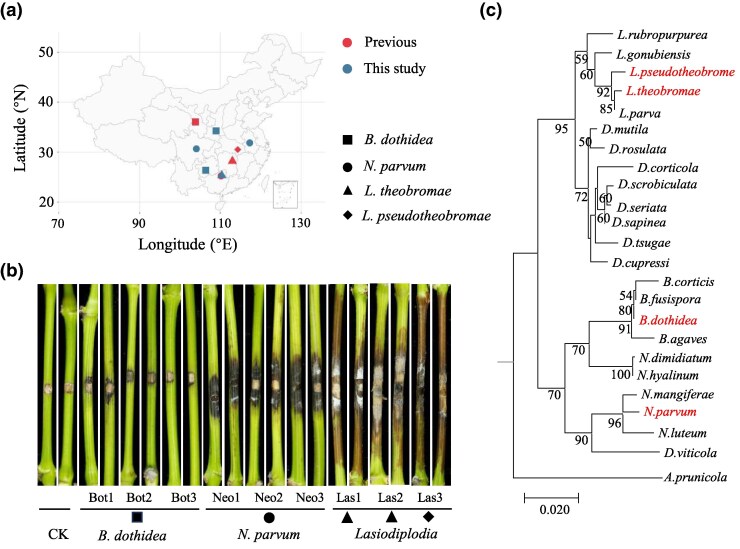


instead of:

**Figure:**
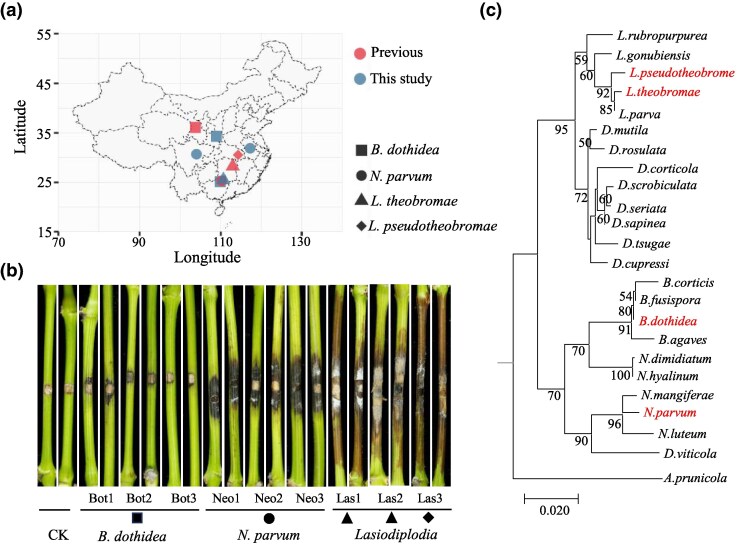


and in panel a of Supplementary Figure S2 which should now read:

**Figure:**
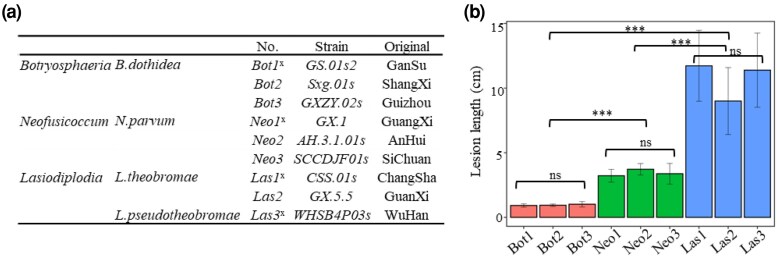


instead of:

**Figure:**
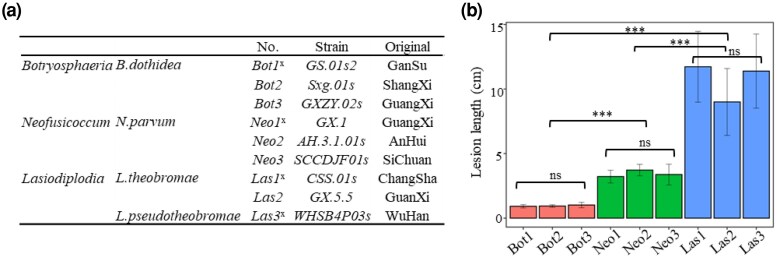


In addition, while preparing the updated Figure 1a, the geographic background of the panel was corrected and the coordinate-axis labels as **“Latitude (°N)”** and **“Longitude (°E)”**. These changes are graphical only.

These corrections are limited to the displayed sampling-location information and figure presentation. The sampling location was not used in any downstream analyses in the study and its correction does not affect the strain identity, experimental data, genome analyses, phylogenetic analyses, gene family analyses, statistical results, interpretations, or conclusions of the article.

