# Supplementary material for: Lineage-Specific Gene Family Innovations Underpin the Pathogenicity of Woody-Plant Pathogens in Botryosphaeriaceae
Source: Genome Biol Evol. 2026 Jul 17;18(7):evag178. doi: 10.1093/gbe/evag178 (PMC13403568; doi:10.1093/gbe/evag178)
Supplement: evag178_Supplementary_Data [file evag178_supplementary_data.zip › SupFigures.pdf]

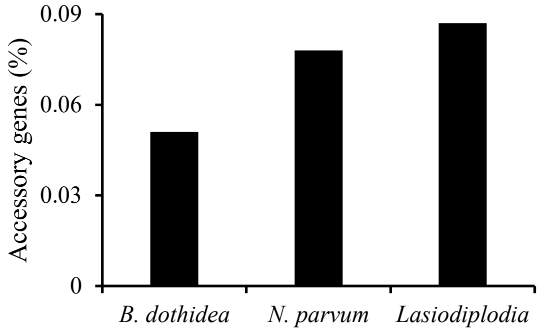

Fig. S1. Accessory protein variation within Botryosphaeriaceae strains analyzed in this study. Proportion of accessory proteins within each group of three strains: *B. dothidea* (3 strains), *N. parvum* (3 strains), and *Lasiodiplodia* (3 strains, comprising two species). Accessory proteins are defined as proteins present in only one or two of the three strains within each group.

(a)

		No.	Strain	Original
Botryosphaeria	*B.dothidea*	*Bot1*ˣ	*GS.01s2*	GanSu
		Bot2	*Sxg.01s*	ShangXi
		Bot3	*GXZY.02s*	GuangXi
Neofusicoccum	*N.parvum*	*Neo1*ˣ	*GX.1*	GuangXi
		Neo2	*AH.3.1.01s*	AnHui
		Neo3	*SCCDJF01s*	SiChuan
Lasiodiplodia	*L.theobromae*	*Las1*ˣ	*CSS.01s*	ChangSha
		Las2	*GX.5.5*	GuanXi
	L.pseudotheobromae	*Las3*ˣ	*WHSB4P03s*	WuHan

(b)

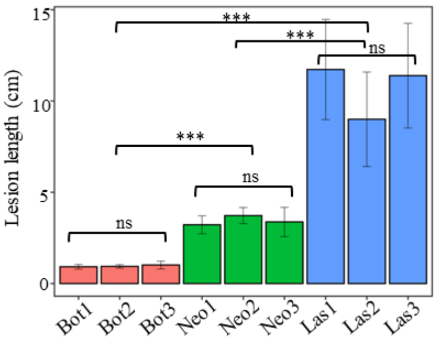

Fig. S2. Virulence differences among Botryosphaeriaceae strains on grapevine shoots. a) Table summarizing information for the nine strains. "No." indicates the strain IDs assigned in this study, "Strain" shows the original names, and "Origin" indicates the sampling location. An "X" in the "No." column indicates that the genome of the corresponding strain was previously sequenced and assembled (Wang et al., 2024). b) Lesion length comparison among nine strains on grapevine shoots at 10 days post-inoculation. Each strain included nine biological replicates; error bars represent the standard error. One-way ANOVA showed no significant differences among strains within the Bot (*B. dothidea*, $F = 2.31$, $P = 0.120$), Neo (*N. parvum*, $F = 2.47$, $P = 0.105$), or Las (*Lasiodiplodia*, $F = 1.87$, $P = 0.176$) groups, with Tukey's HSD tests confirming no pairwise differences. However, highly significant differences were detected among the Bot, Neo, and Las groups ($F = 287.4$, $P < 2.2 \times 10^{-16}$), and all pairwise group comparisons were significant (Tukey's HSD post-hoc tests, $P < 2 \times 10^{-16}$). ***: $P < 0.001$; ns: not significant.

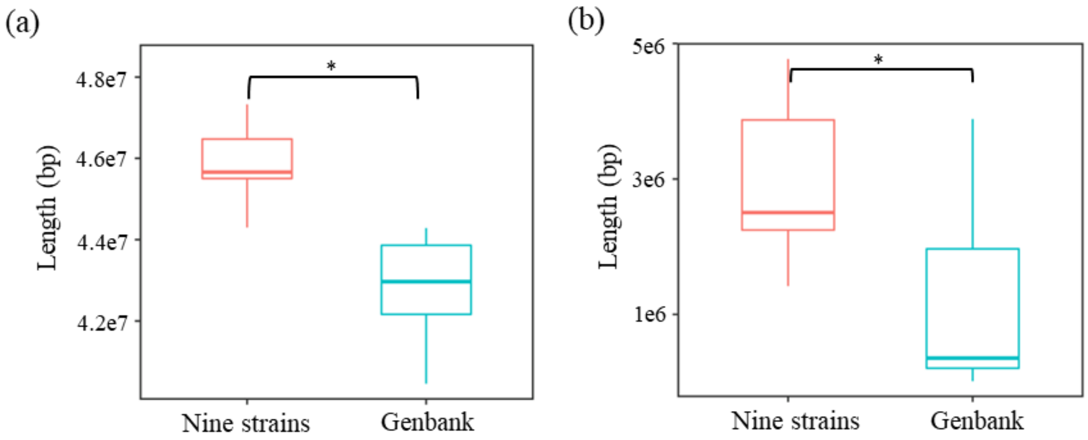

Fig. S3. Comparison of genome quality between the nine Botryosphaeriaceae strains analyzed in this study and additional genomes from GenBank. (a) Genome size ($W = 79$, $P = 0.012$). (b) N50 values ($W = 79$, $P = 0.012$). The nine strains represent the genomes generated in this study, whereas the GenBank dataset includes 11 publicly available Botryosphaeriaceae genomes: *B. dothidea* (5), *N. parvum* (3), and *L. theobromae* (3). Statistical significance was assessed using the Wilcoxon rank-sum test. *: $P < 0.05$.

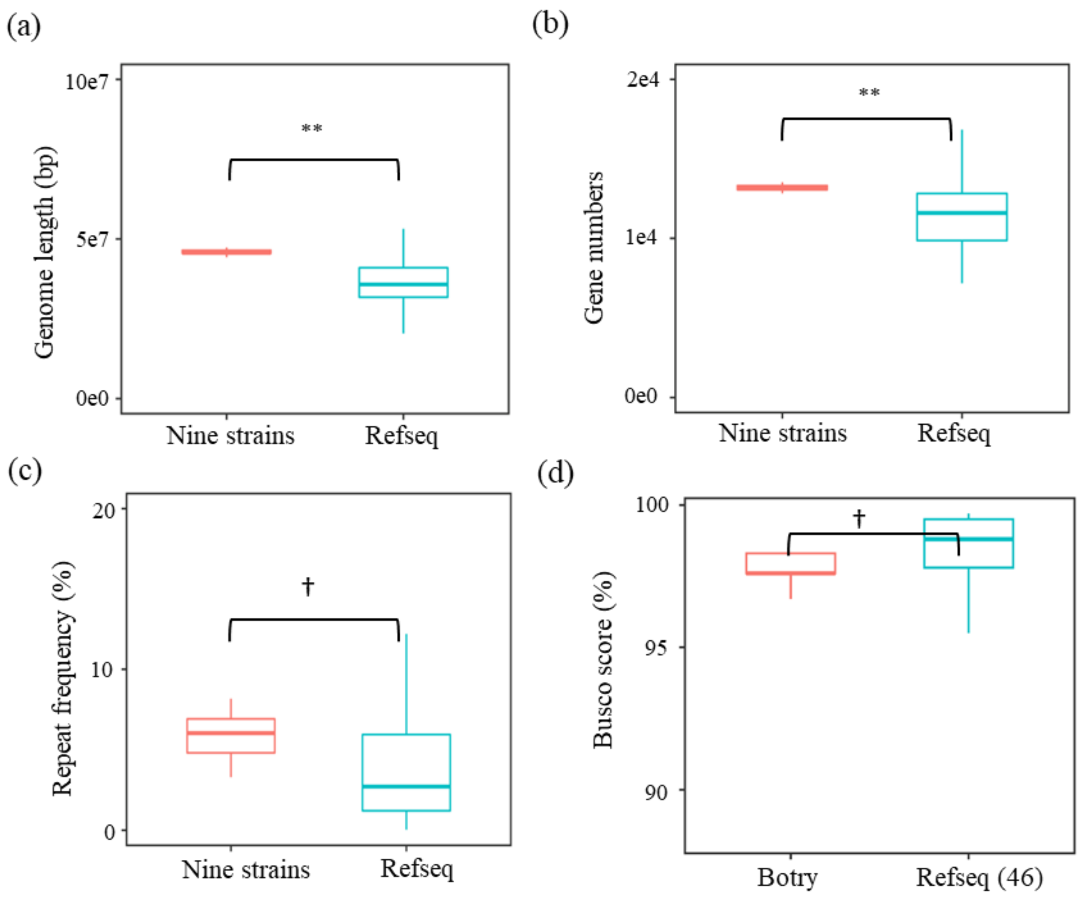

Fig. S4. Comparison of genome features between Botryosphaeriaceae strains and other Ascomycota species from the RefSeq database. a) Genome size (W = 1250, P = 0.001). b) Gene number (W = 1211, P = 0.003). c) Repeat content (W = 1119, P = 0.07). For panels (a–c), genome features of the nine strains used in this study were compared with those of 504 Ascomycota species obtained from the RefSeq database. d) Comparison of protein BUSCO completeness between the 10 Botryosphaeriaceae genomes shown in Fig. 2a and 46 representative fungal genomes from RefSeq (W = 145, P = 0.06). Statistical significance was assessed using the Wilcoxon rank-sum test. **: $P < 0.01$; †: $P < 0.1$.

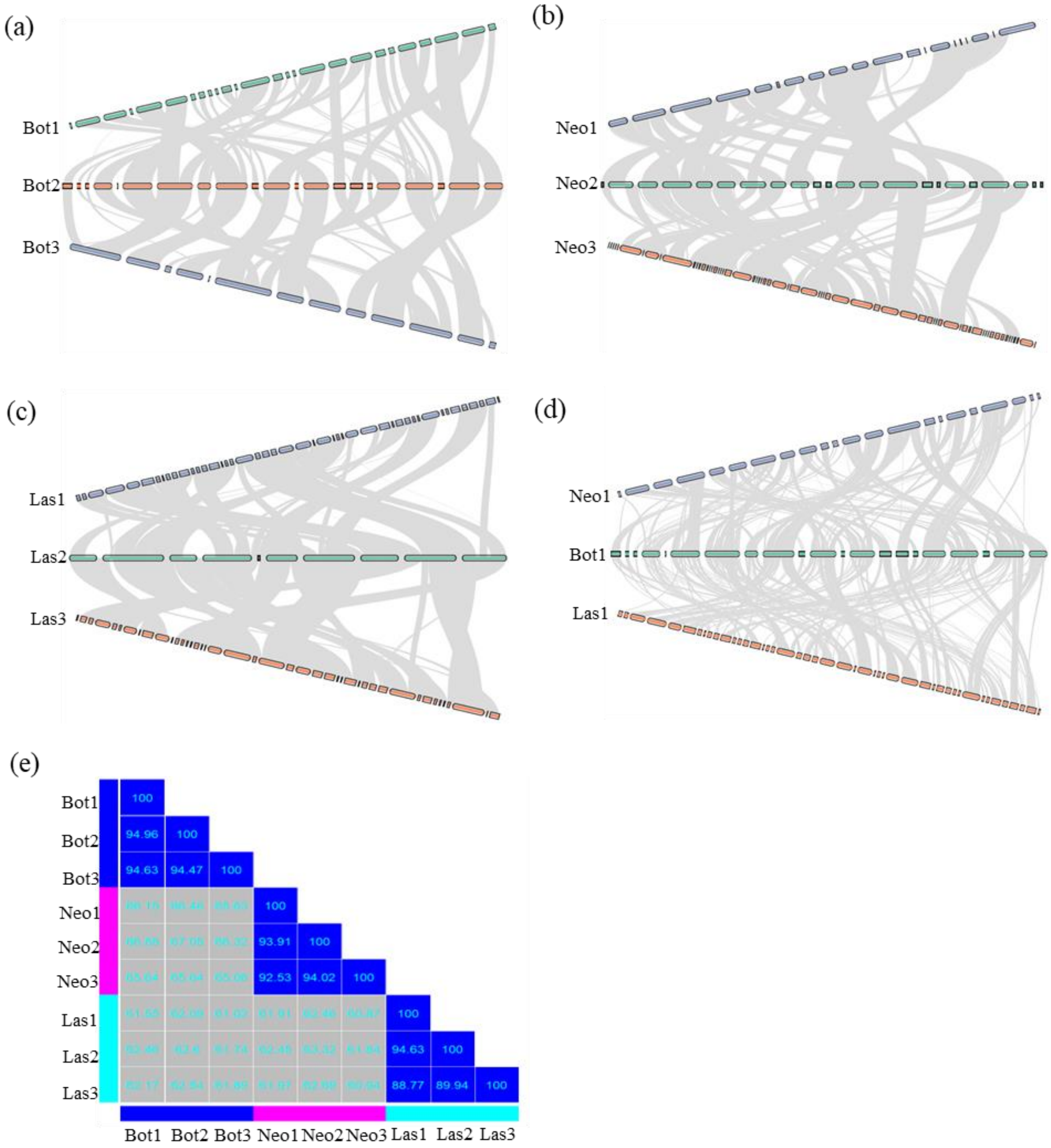

Fig. S5. Genome collinearity among the nine Botryosphaeriaceae strains. a) Collinearity among the three *B. dothidea* strains. b) Collinearity among the three *N. parvum* strains. c) Collinearity among the three *Lasiodiplodia* strains. d) Collinearity between strains from different genera. e) Proportion of collinear genes (%) between pairwise genome comparisons of the nine strains. For each strain in a row, the proportion of collinear genes was calculated relative to the strain shown in the corresponding column.

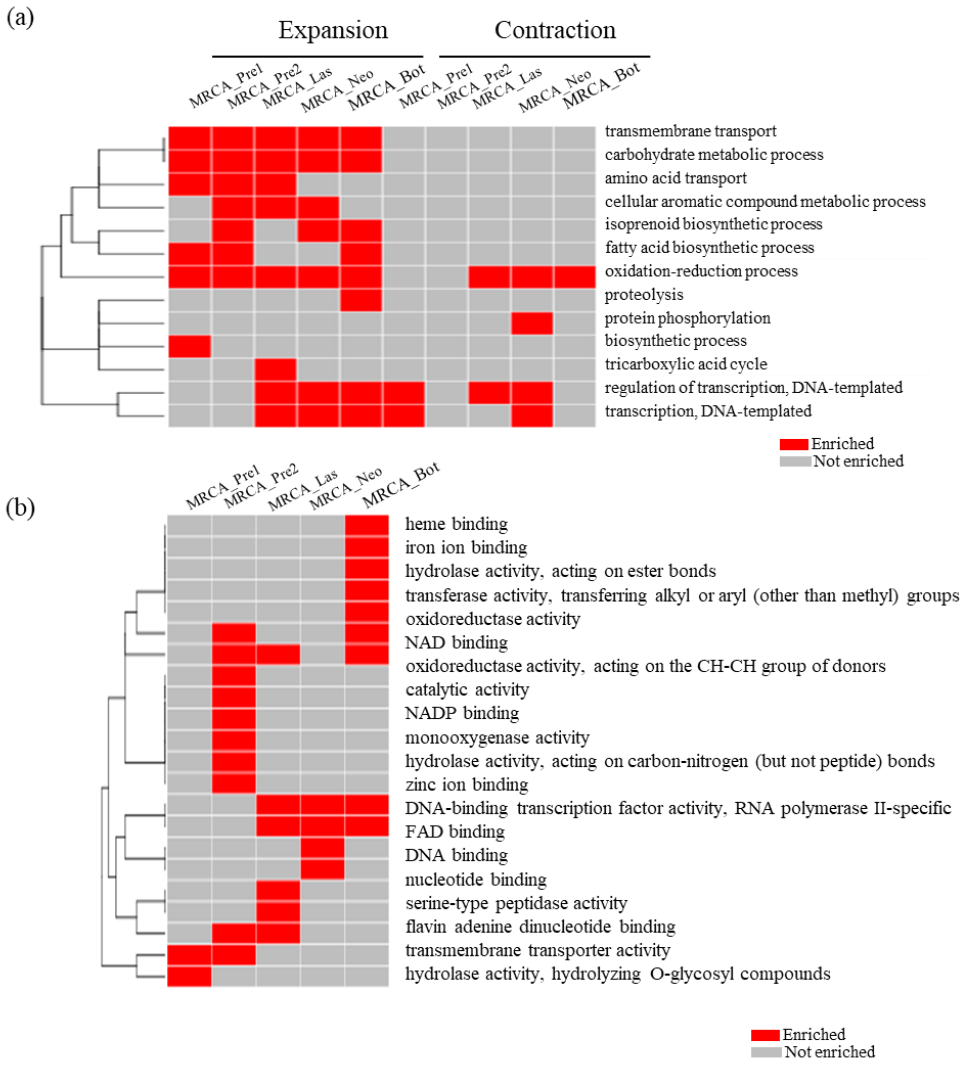

Fig. S6. Gene ontology analysis of gene family expansion and contraction in Botryosphaeriaceae. a) GO enrichment analysis of expanded and contracted gene families in Botryosphaeriaceae. b) GO enrichment analysis of lineage-specific expanded gene families at the five MRCA nodes shown in Fig. 2c. The heatmap shows the enrichment status of GO terms across ancestral nodes. Red indicates significantly enriched terms (*FDR* < 0.05), and grey indicates non-significant terms.

	CAZyme	Effector	Protease	SM	P450	Transporter	TF
B.zeicola	492	239	432	47	146	707	383
B.victoriae	503	266	443	44	140	702	404
B.oryzae	491	220	427	38	127	687	377
B.maydis	494	237	415	51	153	710	389
B.sorokiniana	489	227	422	40	131	695	413
E.turcica	459	200	368	46	134	682	400
P.tritici-repentis	442	209	353	40	105	680	321
A.alternata	559	215	495	32	127	766	445
A.arborescens	564	246	505	28	124	769	365
L.maculans	445	172	304	28	65	630	283
P.nodorum	459	241	399	33	134	605	263
D.symphoricarpi	417	118	336	30	87	646	333
D.exigua	464	132	367	20	85	678	414
M.anomochaeta	485	151	379	21	77	712	410
T.pertusa	551	232	497	43	191	834	433
P.sporulosa	614	248	556	41	149	819	473
W.ornata	352	147	281	29	68	589	274
A.arxii	566	232	472	41	138	837	564
L.ingoldianus	505	200	428	58	156	795	371
M.resinicola	308	104	257	35	145	800	502
P.hyperparasitica	360	93	289	40	109	660	333
Neo3	556	147	526	62	241	828	450
Neo1	552	143	533	63	262	836	443
Neo2	557	130	518	59	263	834	445
Bot1	552	161	551	70	228	830	461
Bot2	545	155	556	67	275	838	456
Bot3	559	152	537	70	248	842	449
Las1	508	139	494	53	178	819	463
Las2	517	143	501	53	173	818	452
Las3	525	136	491	50	197	818	476
D.corticola	440	72	382	38	141	715	356
A.prunicola	372	137	267	38	93	663	344
C.apollinis	248	77	164	15	82	655	272
E.bilateralis	250	77	211	12	65	609	283
V.gallopava	242	27	157	14	74	688	374
S.musiva	294	100	211	31	75	620	281
C.beticola	424	178	390	61	123	762	379
P.fijiensis	367	113	227	25	96	707	301
Z.cellare	539	174	506	44	175	902	570
Z.tritici	360	190	246	30	78	671	249
D.aciculare	213	118	170	31	74	576	262
N.acidophila	213	49	145	13	60	650	323
B.panamericana	289	39	156	15	55	625	267
A.subglaciale	421	81	250	27	72	718	385
A.pullulans	457	93	287	27	62	752	394
A.namibiae	440	59	245	20	59	725	366
C.fructicola	799	547	833	90	283	949	704
F.graminearum	536	224	426	44	112	786	501
N.crassa	372	97	237	19	41	595	285
P.oryzae	550	411	471	53	135	668	354
B.cinerea	517	84	362	50	132	703	406
A.fumigatus	481	76	281	37	75	712	408
A.flavus	557	199	406	72	158	830	549
A.nidulans	493	57	324	53	118	718	488
S.cerevisiae	113	8	34	4	2	446	134
S.pombe	149	11	56	3	3	560	182

Fig. S7. Comparative analysis of pathogenicity-related genes in Botryosphaeriaceae and other fungi. The nine Botryosphaeriaceae strains contain relatively high numbers of genes associated with plant pathogenic lifestyle-related functions, including CAZymes (t = 5.56, P = 1.28 × 10^{-6}), proteases (t = 5.52, P = 1.52 × 10^{-6}), SM (t = 9.99, P = 3.42 × 10^{-13}), cytochrome P450s (t = 7.40, P = 2.15 × 10^{-9}), transporters (t = 6.26, P = 6.45 × 10^{-8}), and transcription factors (TFs) (t = 2.02, P = 0.052) compared with other fungi (columns with red numbers), whereas no significant difference was observed for effector genes (t = -0.12, P = 0.91; green). Welch's t-test with Bonferroni correction was used for statistical analysis. Compared with *Lasiodiplodia*, the genomes of *B. dothidea* and *N. parvum* contain significantly higher copy numbers of CAZymes (t = 6.43, P = 0.002), proteases (t = 5.38, P = 0.004), SM (t = 5.87, P = 0.003), cytochrome P450s (t = 6.02, P= 0.002), and transporters (t = 7.91, P = 0.001), whereas no significant differences were detected for TFs (t = -0.81, P = 0.452) or effector genes (t = 1.82, P = 0.124).

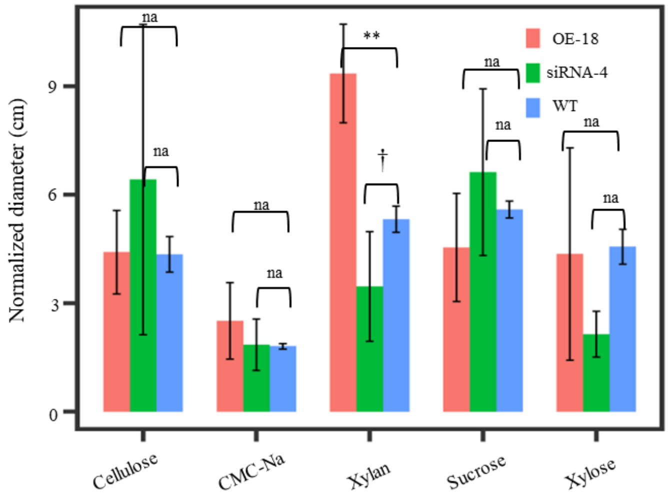

Fig. S8. Relative growth rates of *Lasiodiplodia* strains grown on media with different sole carbon sources. Growth rates of the wild-type (WT), overexpression (OE), and siRNA-silenced (RNAi) strains were calculated from colony diameters on media containing individual carbon sources relative to those on basal medium after incubation at 25 °C for 72 h. Statistical comparisons (OE vs. WT and RNAi vs. WT) for each carbon source were performed using Welch's *t*-test with Bonferroni correction. A significant difference was detected for OE vs. WT on xylan ($t = 5.47$, $P = 0.002$), and a marginal trend was observed for RNAi vs. WT on xylan ($t = -2.22$, $P = 0.078$). *: $P < 0.01$; †: $P < 0.1$; na: $P > 0.1$.

Supplemental tables

Table S1: Summary of sequencing reads generated for nine Botryosphaeriaceae strains.

Table S2: Genome survey of five Botryosphaeriaceae strains using Jellyfish and GenomeScope.

Table S3: Genome lengths of Botryosphaeriaceae strains assembled using different methods.

Table S4: BUSCO completeness scores of five Botryosphaeriaceae genomes assembled using different methods.

Table S5: Mapping rate of Illumina reads to the genomes of Botryosphaeriaceae genomes assembled using different methods.

Table S6: Summary of high-quality genomes assembled for five Botryosphaeriaceae strains.

Table S7: List of GenBank accessions for Botryosphaeriales sequences used in this study.

Table S8. Taxonomic classification of 47 Ascomycota genomes from the RefSeq database.

Table S9. Mapping statistics of RNA-seq reads in Las1.

Table S10. Gene family functional enrichment analysis for families with increased or decreased copy numbers in *Lasiodiplodia* relative to *B. dothidea* and *N. parvum*.

Table S11: Primers used for functional analysis of g11806.t1 in Las1
